# Effect of Seasonal Variations on Soil Microbial, Extracellular Enzymes, and Ecological Stoichiometry in Tea Plantations

**DOI:** 10.1002/ece3.71362

**Published:** 2025-05-12

**Authors:** Tianyi Pu, Yusha Tan, Yuanqi Zhao, Zhibin Zhao, Ni Zhang, Can Li, Yuehua Song

**Affiliations:** ^1^ School of Karst Science Guizhou Norml University/State Engineering Technology Institute for Karst Desertification Control Guiyang Guizhou China; ^2^ Guizhou Provincial Key Laboratory for Rare Animal and Economic Insect of the Mountainous Region Guiyang University Guiyang Guizhou China

**Keywords:** Karst, seasonal variations, soil ecological stoichiometry, soil extracellular enzymes, soil microbes

## Abstract

Tea plantations are important agricultural ecosystems in karst areas, yet the seasonal dynamics of soil microbial communities, functional genes, and extracellular enzyme activities (EEA) under different management practices remain poorly understood. This study investigated organic (HY), pollution‐free (TS), and conventional (XY) tea plantations in Weng'an County, Southwest China, during the spring (April) and autumn (August) tea seasons via metagenomics and stoichiometric analyses. Seasonal variations significantly altered the soil physicochemical properties (e.g., SOC, TN, and TP) and EEA (*p* < 0.05), with higher C‐acquiring enzyme activity in autumn and elevated soil C:N:P ratios in spring. The soil extracellular enzyme stoichiometry (EES C:N:P) deviated from the theoretical 1:1:1 ratio, indicating that microbial metabolism was constrained by soil resource availability rather than homeostasis. Phosphorus limitation (vector angle > 45°) persisted across seasons, contradicting initial hypotheses, with acid phosphatase (ACP) activity and EES C:P identified as critical drivers. Random forest (RF) and structural equation modeling (SEM) revealed that the spring season had stronger impacts on microbial communities and functional genes, with the soil TN, C:N, NAG, ACP, and EES C:P ratios as key predictors. Compared with conventional practices, organic management enhances microbial diversity and functional redundancy, buffering seasonal fluctuations. These findings highlight the interplay between seasonal climatic shifts and agricultural practices regulating soil nutrient cycling and microbial adaptation. Strategic interventions—such as spring carbon supplementation, autumn organic phosphorus fertilization, and intercropping—are proposed to optimize microbial resilience and ecosystem stability in fragile karst tea plantations. This study provides novel insights into soil ecological stoichiometry and microbial metabolic strategies, offering a reference for the sustainable management of agroecosystems in karst areas.

## Abstract 2

Random forest model and constructive equation model were used to analyze the soil environmental factors of mountain tea plantations in karst areas during the spring and autumn tea seasons. Soil extracellular enzyme activities of tea plantations in different seasons were significantly higher in autumn than in spring. Soil microbial richness and diversity in tea plantations increased significantly during the autumn tea season. Functional genes for carbon fixation, nitrogen cycling, and phosphorus cycling in tea plantation soils respond differently to seasonal changes. Greater influence of soil environmental factors on soil microorganisms and soil functional genes during the spring tea season.

## 1 Introduction

Tea tree ( *Camellia sinensis* ), a vital cash crop in southern China with a history of cultivation for centuries (Yan et al. 2018), has undergone extensive cultivation practices that may induce soil acidification. Such acidification significantly alters nutrient availability (Yan et al. 2020; Yi, Zeng, et al. 2022), compelling microbial communities to adapt through enzyme production adjustments. These adaptations threaten the sustainability of tea plantation ecosystems (Wang et al. 2025). Nevertheless, current research has focused predominantly on natural vegetation or traditional farmland, resulting in a poor understanding of soil microorganisms in tea plantations under different agricultural management practices.

Soil carbon (C), nitrogen (N), and phosphorus (P) are fundamental drivers of biogeochemical cycles in agroecosystems. Their stoichiometric ratios (C:N:P) regulate nutrient availability and microbial metabolic constraints, with imbalances potentially destabilizing ecosystem functioning (Sinsabaugh et al. 2009; Zhou et al. 2017). In tea plantations, soil nutrient cycling, a cornerstone of soil fertility and crop productivity, is modulated by interactions between abiotic factors, such as temperature and moisture, and management practices (Tang and Huang 2009). Critically, agricultural interventions such as fertilization alter not only individual nutrient availability but also the coupled biogeochemical cycles of C, N, and P (Bai et al. 2018). For example, nitrogen addition may exacerbate phosphorus limitation by disrupting microbial enzyme allocation (Cui et al. 2020). These interdependencies underscore the need for stoichiometrically informed management strategies to sustain tea plantation ecosystems.

Soil microorganisms are the linchpins of agroecosystem functionality. They drive organic matter decomposition, humus formation, and nutrient cycling–transformation processes (Lori et al. 2023; Leff et al. 2015). Crucially, even in nutrient‐poor environments, microbial communities adapt by modulating extracellular enzyme production to increase nutrient acquisition, thereby sustaining community‐level nutrient homeostasis (Wang, Li, et al. 2023). These adaptive strategies are quantifiable through two key metrics: soil nutrient stoichiometry (C:N:P) and extracellular enzyme stoichiometry (EES C:N:P). The former reflects elemental balances in soil resources, whereas the latter directly indicates microbial nutrient acquisition priorities and metabolic constraints (Zhou et al. 2017). While some studies report EES homeostasis (C:N:P≈1:1:1) as a strategy to balance resource demands (Bastida et al. 2016; Loeppmann et al. 2016; Ye et al. 2022), others demonstrate substrate‐dependent EES dynamics in variable resource environments (Peng and Wang 2016). Karst tea plantations, which are subjected to high management intensity and seasonal climatic fluctuations, present an ideal system to test these competing hypotheses. Their inherently fragile habitats, coupled with high management intensity, impose dual pressures on microbial communities. However, whether microbial EES strategies in these systems align with global models remains unresolved, hindering predictions of microbial metabolic efficiency under changing resource regimes (Yi, Ji, et al. 2022; Yi, Zeng, et al. 2022).

Seasonal dynamics drive soil microclimate formation and plant activity, shaping the microbial community structure (Grayston et al. 2001). They also regulate enzyme production and reaction kinetics, which in turn alter biochemical processes essential for soil functionality (Bell et al. 2014). Notably, compared with other organisms, soil microbial communities are more sensitive to temporal changes and undergo rapid turnover to stabilize their ecological roles (Rousk and Bååth 2011). For example, studies on grassland microenvironments have revealed pronounced seasonal variations in the spatial drivers of microbial community composition and enzyme activity (Wang, Henry, et al. 2023; Wang, Li, et al. 2023). Climate warming exacerbates these effects, intensifying seasonal fluctuations, reducing constraints on microbial populations during the growing season, and disrupting extracellular enzyme stoichiometry (Zheng et al. 2020). Despite progress, most studies have focused on microbial community dynamics and soil extracellular enzyme activities (EEA)/EES, with insufficient exploration of their synergistic relationships (Piotrowska‐Długosz et al. 2019). Given the potential impact of seasonal dynamics on microbial function, clarifying their interaction with different management practices has therefore become a key entry point for this study.

In accordance with the new requirements of modern agricultural business models and sustainable agricultural development, this study analyzed the relationships among soil microbes, soil functional genes, and soil EEA and their response to seasonal variations via metagenomics analysis of Weng'an tea plantations. On the basis of the above background, this study proposes two hypotheses: (H1) seasonal variations (spring vs. autumn) alter the metabolic limitations of soil microbes in karst tea plantations and (H2) organic management enhances ecosystem stability by increasing functional gene diversity and enzyme stoichiometric plasticity, reducing the sensitivity of soil nutrient cycling to seasonal changes compared with conventional practices. By elucidating the seasonal dynamics of soil microbial communities, functional genes, and EES, this study aims to reveal the metabolic adaptation strategies of soil microorganisms in karst tea plantations and provide a theoretical basis for precision collaborative fertilization–microbial regulation management models.

## 2 Materials and Methods

### 2.1 Study Site

**FIGURE 1:**
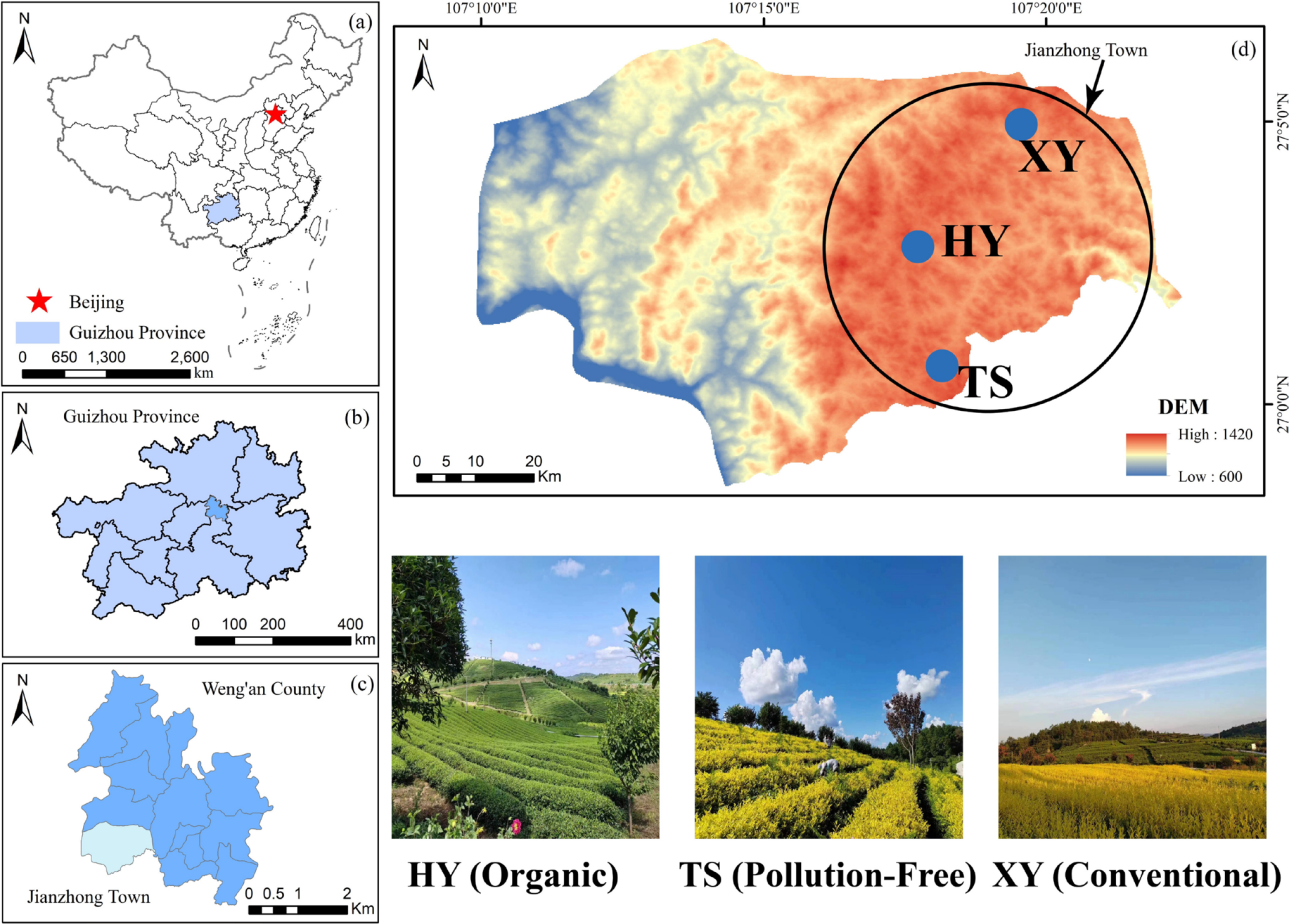
Location map of the study areas.

Weng'an County is in the middle of Guizhou Province, in the middle reaches of the Wujiang River, and in the northernmost part of the Qiannan Buyei and Miao Autonomous Prefectures (107°42′ E, 27°29′ N). It has a subtropical monsoon climate with an average multiyear temperature of 13.9°C and an average multiyear precipitation of 1100 mm (Huang and Ding 2017). The soil distribution is mainly yellow soil developed from sandy shale, which is slightly acidic and suitable for tea tree growth. In the present study, Huanghongyin (HY, Organic), Tianshun (TS, PolFree), and Xinyu (XY, Conv) high mountain tea plantations (altitude > 1000 m) were selected as the study sites (Figure 1). Mature (> 10 years) “Huangjinya” tea trees are cultivated at these sites. “Huangjinya” is a bright yellow tea with a bright yellow soup color and a pure yellow leaf bottom. It has a high amino acid content and a fresh taste, so it is favored by tea consumers and has good economic benefits (Fei and Wang 2014). Because of its excellent quality traits, it has become a protected geographical indication product and has been conferred a geographical indication certification trademark.

### 2.2 Data Collection

**FIGURE 2:**
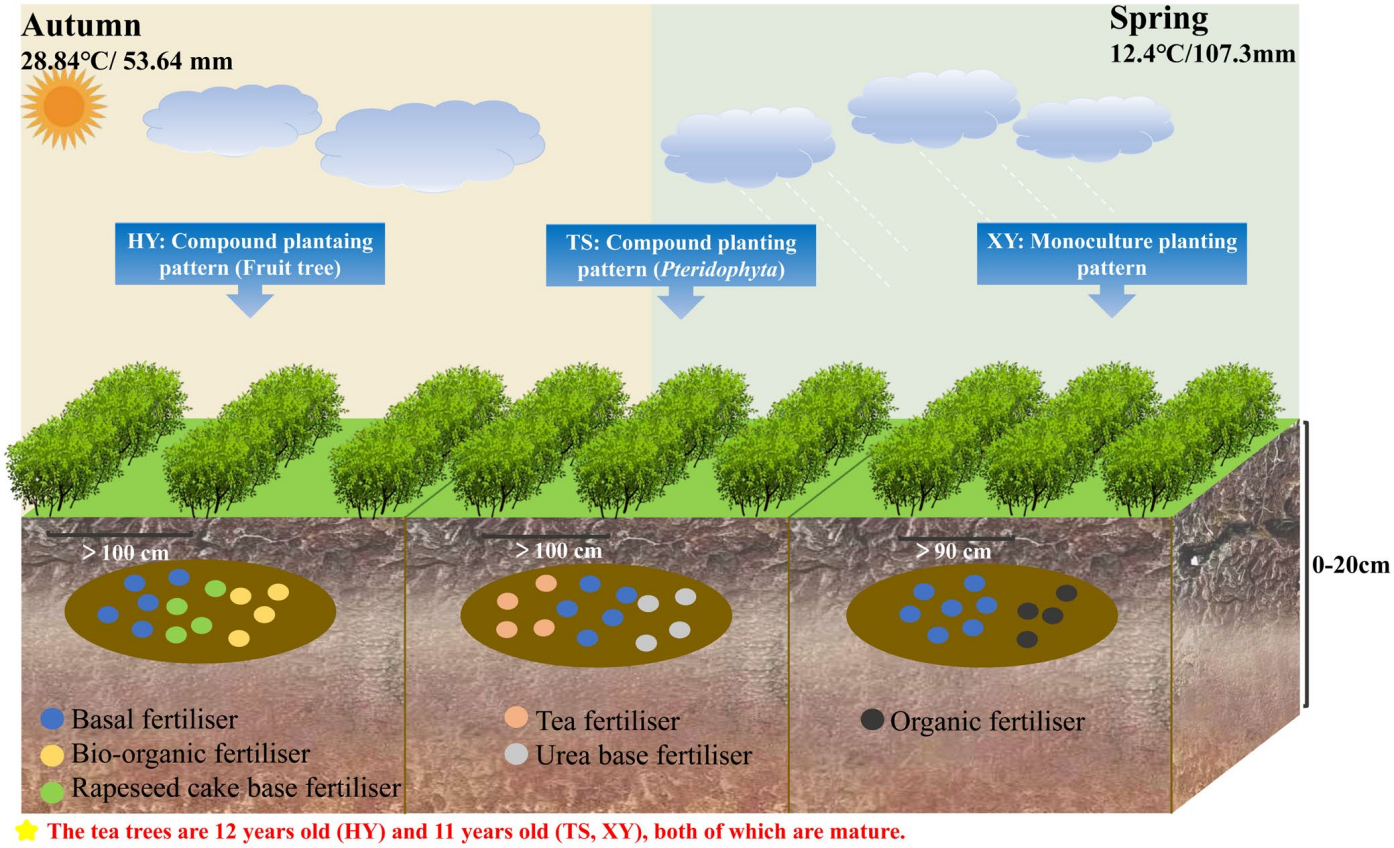
Information map of the research area.

On the basis of the field investigation, soil samples were collected from the study sites HY, TS, and XY in August 2022 and April 2023. There was sunny weather for more than 15 days before sampling, which ensured a low level of soil variability and a relatively low number of tests. The study sites are relatively close to each other and therefore have similar climatic backgrounds and geological conditions. In addition, the study sites are cultivated with the same variety and age of tea trees—“Huangjinya”—so that they are influenced by similar natural factors. The characteristics of the sampling plots are shown in Table S1. Five 10 × 10 m sample squares (with sufficient buffer strips between squares) were set up in each sampling plot. Multiple sampling points were laid along S‐shaped lines in the sample squares, and at each sample point, equal amounts of soil obtained from 0 to 20 cm soil layers (< 20 cm from the actual depth), mixed evenly, were collected. This occurred because the 20 cm depth is the main area in which the root system of the tea tree is concentrated, and 25 soil samples were collected (Figure 2, Table S1). After removing stones and debris, the samples were stacked according to the diagonal method until the sample weight reached the value required to establish a composite sample. The collected samples were divided into two parts: one was used to determine their physicochemical properties, and the other was used for molecular experiments (sealed at −80°C for DNA extraction).

### 2.3 Determination of the Physical and Chemical Properties of Soils

The soil organic carbon (SOC) content was determined via the potassium dichromate external heating method. The molybdenum‐antimony anti‐colorimetric method was used to determine total protein (TP) content. The total nitrogen (TN) content was determined via an automatic Kjeldahl apparatus (Shandong Haineng Scientific Instrument, Shandong, China).

### 2.4 Determination of Soil Extracellular Enzyme Activity

The potential activities of two C‐acquiring enzymes (β‐1,4‐glucosidase, βG; cellobiohydrolase, CBH), two N‐acquiring enzymes (β‐1,4‐N‐acetylglucosaminidase, NAG; leucine aminopeptidase, LAP), and one P‐acquiring enzyme (acid phosphatase, ACP) were determined via fluorometric assays according to the protocols proposed by Saiya‐Cork et al. (2002). The enzyme activities were measured using 1 g of fresh soil, which was placed into 125 mL of deionized water and homogenized for 2 h at 180 r min^−1^ on a rotary shaker (25°C). Next, 200 μL of each sample was placed into a 96‐well microplate (six parallel subsamples for each sample) before 50 μL of the 200 μmol L^−1^ substrate was added. In addition, 50 μL of deionized water and 200 μL of each sample were placed into blank microplates. We added 50 μL of standard substrate (4‐MUB at 10 μmol L^−1^ for βG, CBH, NAG, and ACP and 10 μmol L^−1^ 7‐amino‐4‐methylcoumarin for LAP) and 200 μL of sample suspension to the quenched standard microplates. The negative control microplates contained 50 μL of substrate and 50 μL of deionized water. We added 50 μL of standard material and 200 μL of deionized water to the microplates. The blank and negative control for each sample comprised three parallel samples. The quenching standard and reference standard comprised six parallel samples for each microplate. The 96‐well microplates were incubated in the dark at 25°C for 4 h. The 4‐MUB fluorescence excitation and detection wavelengths were 365 and 450 nm, respectively (German et al. 2011).

### 2.5 Quantification of Microbial Metabolic Limitation

The stoichiometry of EES was calculated according to the following equation:

(1) $$\mathrm{EES}\;C:N=\ln\left(\mathrm{\beta G}+\mathrm{CBH}\right)/\ln\left(\mathrm{NAG}+\mathrm{LAP}\right)$$

(2) $$\mathrm{EES}\;C:P=\ln\left(\mathrm{\beta G}\right)/\ln\left(\mathrm{ACP}\right)$$

(3) $$\mathrm{EES}\;N:P=\ln\left(\mathrm{NAG}+\mathrm{LAP}\right)/\ln\left(\mathrm{ACP}\right)$$

The microbial metabolic limitation values were quantified by calculating the vector length and angle for enzyme activity on the basis of the untransformed proportional activity (Moorhead et al. 2016). The vector length represents microbial C limitation: lengths indicate stronger C limitations. A vector angle < 45° indicates microbial N limitation, whereas an angle > 45° denotes P limitation. The vector length and angle are calculated via Equations (4) and (5):

(4) $$\text{Vector length}=\text{SQRT}\left(\left(\frac{\ln\left(\mathrm{\beta G}+\mathrm{CBH}\right)}{\ln\left(\mathrm{ACP}\right)}\right)+\left(\frac{\ln\left(\mathrm{\beta G}+\mathrm{CBH}\right)}{\ln\left(\mathrm{NAG}+\mathrm{LAP}\right)}\right)\;\right)$$

(5) $$\text{Vector angle}=\text{Degrees}\left[\text{ATAN}2\frac{\ln\left(\mathrm{\beta G}+\mathrm{CBH}\right)}{\ln\left(\mathrm{ACP}\right)},\frac{\ln\left(\mathrm{\beta G}+\mathrm{CBH}\right)}{\ln\left(\mathrm{NAG}+\mathrm{LAP}\right)}\;\right]$$

### 2.6 DNA Extraction and Determination of Soil Microbial Metagenomic Amplicons

Total genomic DNA was extracted from fresh soil samples (0.5 g) via the E.Z.N.A. Soil DNA Kit (Omega Biotek, Norcross, GA, U.S.) following the manufacturer's instructions. The concentration and purity of the extracted DNA were determined via TBS‐380 and NanoDrop2000, respectively. DNA extraction quality was evaluated via 1% agarose gel electrophoresis. The DNA extract was fragmented to an average size of approximately 400 bp via a Covaris M220 (Gene Company Limited, China) for paired‐end library construction. A paired‐end library was constructed via NEXTFLEX Rapid DNA‐Seq (Bioo Scientific, Austin, TX, USA). Adapters containing the full complement of the sequencing primer hybridization sites were ligated into the blunt ends of the fragments. Paired‐end sequencing was performed on an Illumina NovaSeq/HiSeq Xten (Illumina Inc., San Diego, CA, USA) at Majorbio Bio‐Pharm Technology Co. Ltd. (Shanghai, China) via NovaSeq Reagent Kits/HiSeq X Reagent Kits following the manufacturer's instructions (www.illumina.com). The data were analyzed on the free online Majorbio Cloud Platform (www.majorbio.com). Paired‐end Illumina reads were trimmed from adaptors, and low‐quality reads (length < 50 bp, quality value < 20, or having *N* bases) were removed via fastp (https://github.com/OpenGene/fastp, version 0.20.0). The metagenomic data were assembled via MEGAHI (https://github.com/voutcn/megahit, version 1.1.2), which uses succinct de Bruijn graphs. Contigs with a length ≥ 300 bp were selected as the final assembly result and then used for further gene prediction and annotation.

### 2.7 Bioinformatics Analysis and Taxonomic Annotation

Fastp (https://github.com/OpenGene/fastp, version 0.20.0) was used to reduce the quality of the adapter sequences at the 3' and 5' ends of the reads, and high‐quality paired‐end reads and single‐end reads were retained. MEGAHIT (Li et al. 2015; version 1.1.2) was used to assemble optimized sequences. Contigs ≥ 300 bp in length were selected as the final assembly results. Prodigal (Hyatt et al. 2010) and MetaGene (Noguchi et al. 2006) (http://metagene.cb.k.u‐tokyo.ac.jp/) were used to predict the ORFs of the contigs. CD‐HIT (Fu et al. 2012) (http://www.bioinformatics.org/cd‐hit/, version 4.6.1) was used to cluster the gene sequences predicted for all samples (parameters: 90% identity, 90% coverage). The longest gene in each class was used as a representative sequence to construct a nonredundant gene set. The high‐quality reads and nonredundant gene sets of each sample were compared via SOA Paligner (Li et al. 2008) software (http://soap.genomics.org.cm/, version 2.21).

Representative sequences of the nonredundant gene catalog were aligned to the NR database with an e‐value cut‐off of 1e‐5 via Diamond (http://www.diamondsearch.org/index.php, version 0.8.35) for taxonomic annotations. The KEGG annotation was conducted via Diamond (http://www.diamondsearch.org/index.php, version 0.8.35) against the Kyoto Encyclopedia of Genes and Genomes database (http://www.genome.jp/keeg/), with an e‐value cut‐off of 1e‐5.

### 2.8 Statistical Analysis

Two‐way analysis of variance (ANOVA) and independent samples t tests were employed to assess differences in tea quality and soil physicochemical properties among tea plantations managed under different practices, followed by the least significant difference (LSD) test (*p* < 0.05). The data were log‐transformed when needed to achieve homogeneity of variance. The composition and diversity of the soil microbial communities in the tea plantations were analyzed. The present study analyzed the effects of different agricultural management practices on the microbial community structure on the basis of nonmetric multidimensional scaling (NMDS) of the Bray–Curtis difference combined with analysis of similarities (ANOSIM). The soil physicochemical properties and soil extracellular enzyme activities were analyzed by the variance inflation factor (VIF), with a VIF < 10, and redundancy analysis (RDA) and Mantel test analysis were performed. The statistical analyses were conducted via the R software packages “vegan” (Oksanen et al. 2013) and “ggcor” (Huang et al. 2020). Data visualization was performed via Origin 2021 (OriginLab, Northampton, MA, USA). We utilized a machine learning algorithm implemented in the randomForest function of the ‘RANDOM FOREST’ package (Liaw and Wiener., Liaw and Wiener 2002) to identify the most important variables. Finally, segmented structural equation modeling (SEM) analysis was conducted via the R package “lavaan” (Rosseel., Rosseel 2012) to assess the relationships among the soil microbial community structure, soil physicochemical properties, and tea quality in tea plantations under different agricultural management practices.

## 3 Results

### 3.1 Variations in Soil Physicochemical Properties and EEA

The results of independent samples t‐tests indicated that seasonal variations had a significant (*p* < 0.05) effect on the soil SOC and TP, while the SOC content was lower in autumn than in spring. Seasonal variations also had a significant (*p* < 0.05) effect on soil βG + CBH (C‐acquiring enzyme) and NAG (N‐acquiring enzymes), and the activities were lower in spring than in autumn (Figure S1, Table S2). The results of two‐factor ANOVA revealed that with the exception of soil βG, agricultural management practices had significant (*p* < 0.05) effects on soil physicochemical properties and soil enzyme activities. In the HY, soil physicochemical content and soil enzyme activities were higher than those of other management patterns, except for SOC and CBH (*p* < 0.05; Figure S1, Table S2).

### 3.2 Variations in Soil Ecological Stoichiometry

Independent sample t tests revealed that the seasonal variations significantly increased the soil C:N and C:P ratios (*p* < 0.05), with higher values observed in spring. However, the N:P ratio showed no significant seasonal differences. The results of two‐factor ANOVA revealed that agricultural management practices also significantly affected the soil C:P ratio, with TS soils exhibiting the highest C:P ratio in spring compared with HY and XY soils (*p* < 0.05).

Table S2 shows that the soil EES exhibited seasonal variations (*p* < 0.05). In spring, the EES C:P and EES N:P ratios were lower than 1, and the soil EES C:N ratio was greater than 1. In autumn, the soil EES C:N ratio remained above 1 (Figure S1, Table S2). The enzyme vector model revealed that seasonal variations significantly affected the vector angle. Most of the vector angles and vector lengths significantly differed (*p* < 0.05) among the tea plantations with different agricultural management practices. The vector angle of the soil enzymes in all the tea plantations was greater than 45°, indicating relative phosphorus limitation of the microbial community. The soil enzyme vector length under HY was lower than that under the other two management practices (Figure S1, Table S2).

### 3.3 Variations in Soil Microorganisms

In this study, the Illumina high‐throughput sequencing platform was used to determine the microbial composition of all the soil samples via metagenomic sequencing technology. The autumn soil samples yielded 778,826,684 raw reads (an average of 51,921,778 reads per sample). The dominant bacterial phyla were Proteobacteria, Actinobacteria, and Acidobacteria; the fungal communities were primarily Mucoromycota, Ascomycota, and Basidiomycota; and the archaeal communities consisted predominantly of Thaumarchaeota, Candidatus Bathyarchaeota, unclassified Archaea, and Euryarchaeota. Spring samples generated 893,491,526 raw reads (an average of 59,566,101 reads per sample), with bacterial dominance shifting to Actinobacteria, Proteobacteria, and Acidobacteria. The fungal and archaeal community compositions remained consistent with the autumn patterns (Figure S2, Table S3).

NMDS and ANOSIM analyses (Bray–Curtis distance) demonstrated significant segregation of microbial communities across management modes (ANOSIM, *R* > 0.5, *p* < 0.05; Figure S3). Seasonal variations and management practices markedly influenced the microbial diversity metrics (*p* < 0.05; Table S4). Specifically, organic management (HY) enhanced bacterial richness (Chao1) and diversity (Shannon index) in autumn (*p* < 0.05). In contrast, pollution‐free management (TS) reduced fungal diversity in autumn (Simpson index). Among the agricultural practices, the composition of the bacterial community varied significantly (ANOVA, *p* < 0.05). Organic management consistently promoted greater microbial richness and diversity than conventional practices (Tables S3 and S4).

### 3.4 Functional Genes Involved in Soil Carbon and Nitrogen Cycling

The functional gene orthologues annotated via the KEGG database revealed 107, 48, and 52 genes associated with carbon fixation (C‐KEGG), nitrogen cycling (N‐KEGG), and phosphorus cycling (P‐KEGG), respectively, in the autumn soil samples compared with 108, 46, and 51 in the spring samples. At Pathway Level 3, the top 5% abundant C‐KEGG pathways included “metabolic pathways, microbial metabolism in diverse environments, carbon metabolism, biosynthesis of secondary metabolites, carbon fixation pathways in prokaryotes, and pyruvate metabolism,” with significantly higher abundance in spring. For N‐KEGG, the dominant pathways (e.g., nitrogen metabolism and biosynthesis of amino acids) presented relatively high abundances in autumn. P‐enriched Kyoto Encyclopedia of Genes and Genomes (KEGG) pathways (e.g., purine metabolism and biosynthesis of secondary metabolites) were enriched in autumn, with the exceptions of ABC transporters and two‐component systems (Figure S4, Table S3).

NMDS and ANOSIM analyses (Bray‐Curtis distance) demonstrated significant differentiation in soil functional gene profiles across management practices (*R* > 0.45, *p* < 0.05). Seasonal variations had no significant impact on N‐KEGG diversity in TS and XY but markedly affected the diversity (Shannon index) and richness (Chao1) of C‐KEGG, N‐KEGG, and P‐KEGG in HY (independent samples *T*‐test, *p* < 0.05). ANOVA indicated significant differences in functional gene composition among management modes during autumn (*p* < 0.05), whereas in spring, only P‐KEGG richness and diversity varied significantly across practices (*p* < 0.05; Table S5, Figure S5).

### 3.5 Linkages Between Microbial Communities, Functional Genes, and Soil Biogeochemical Indicators

**FIGURE 3:**
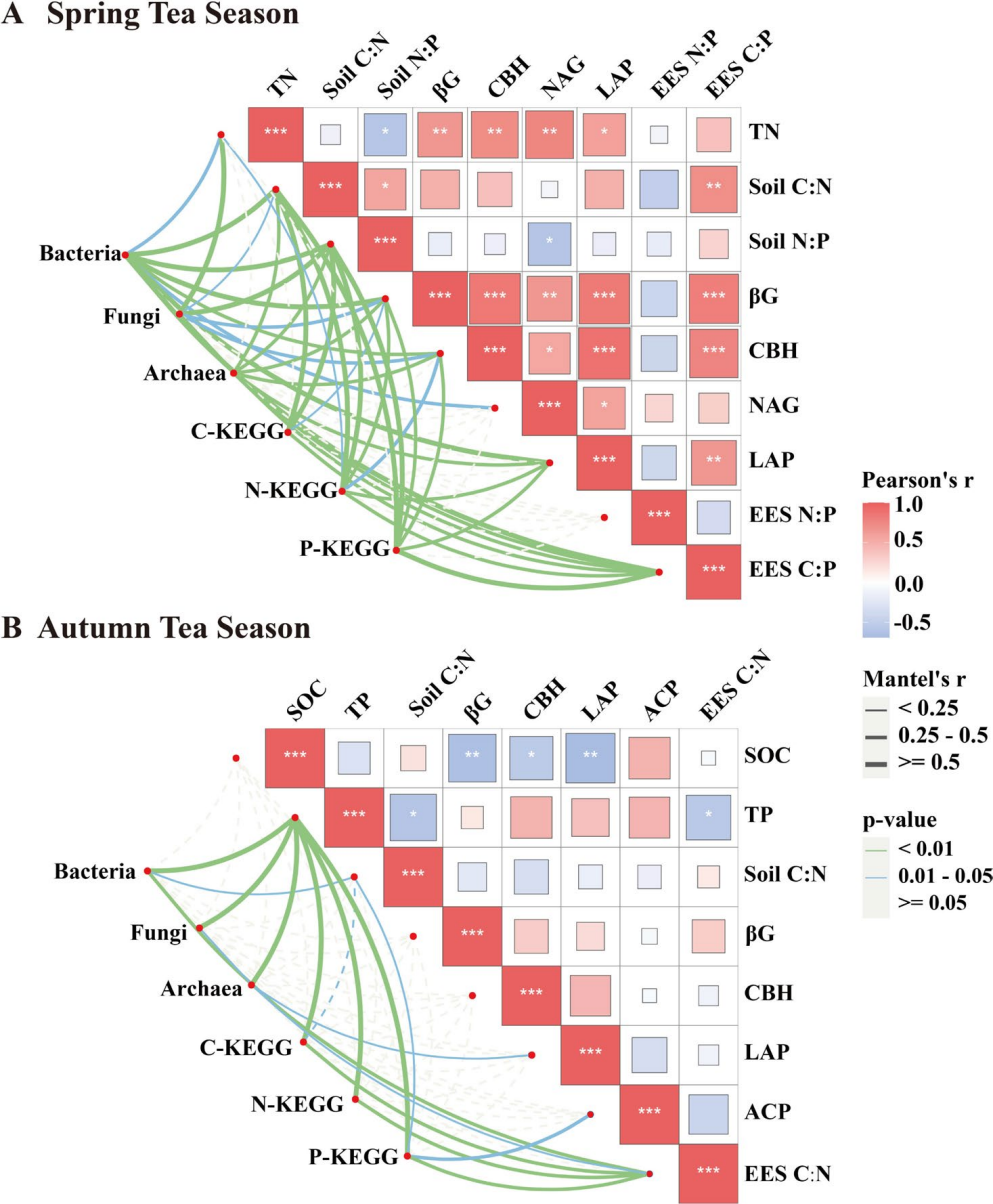
(A, B) are Mantel Test analyses of the association between soil microbial and functional genes, soil physicochemical properties and soil extracellular enzyme activities in tea plantations during early (Spring) and late (Autumn) tea picking (VIF < 10), respectively.

**FIGURE 4:**
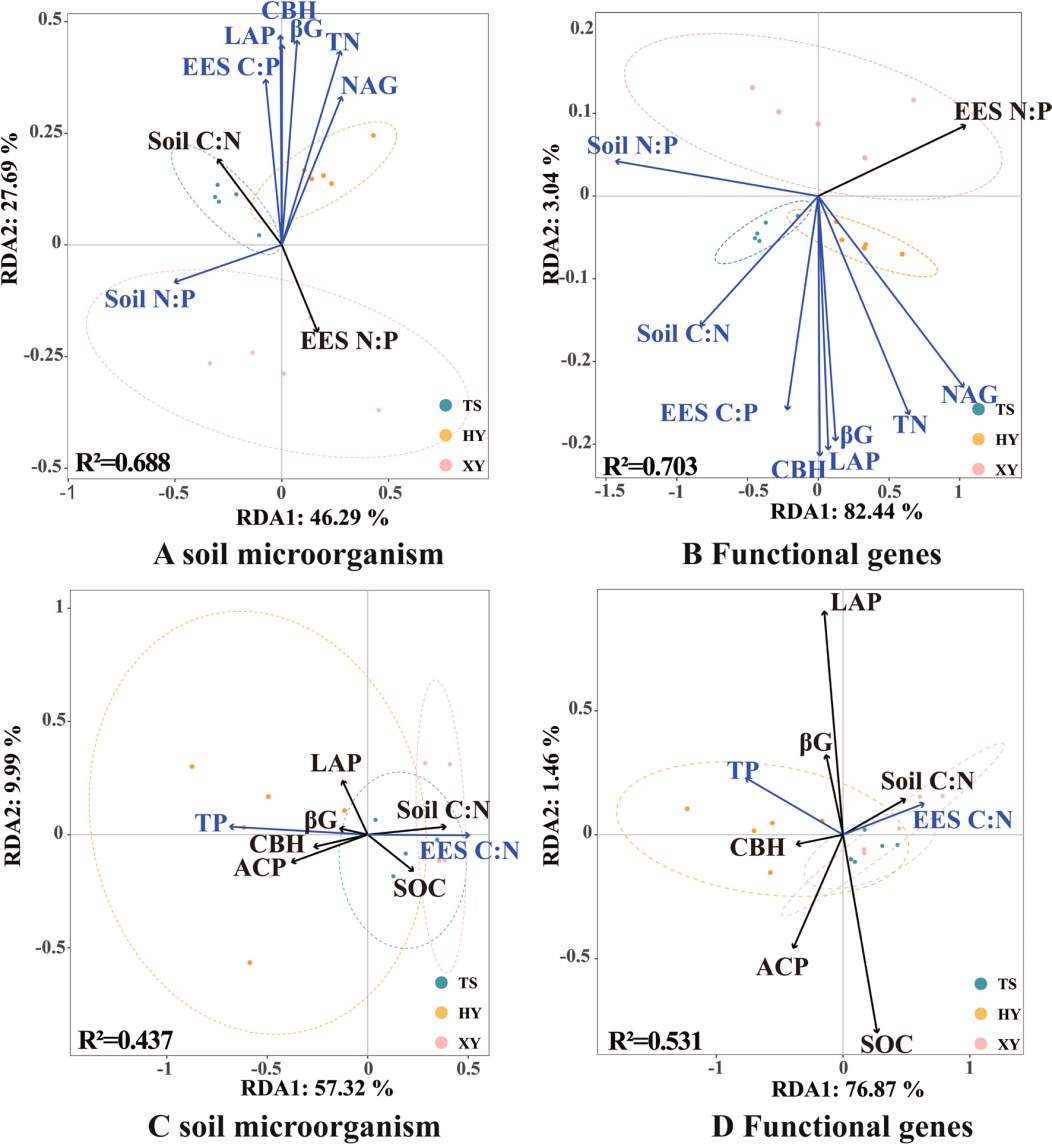
RDA ordination plots of soil microorganisms and functional genes, respectively, in relation to environmental factors, with black lines representing non‐significant correlations and blue lines representing significant correlations. (A,B) are soil microorganisms and soil functional genes in spring. (C,D) are soil microorganisms and soil functional genes in fall.

**FIGURE 5:**
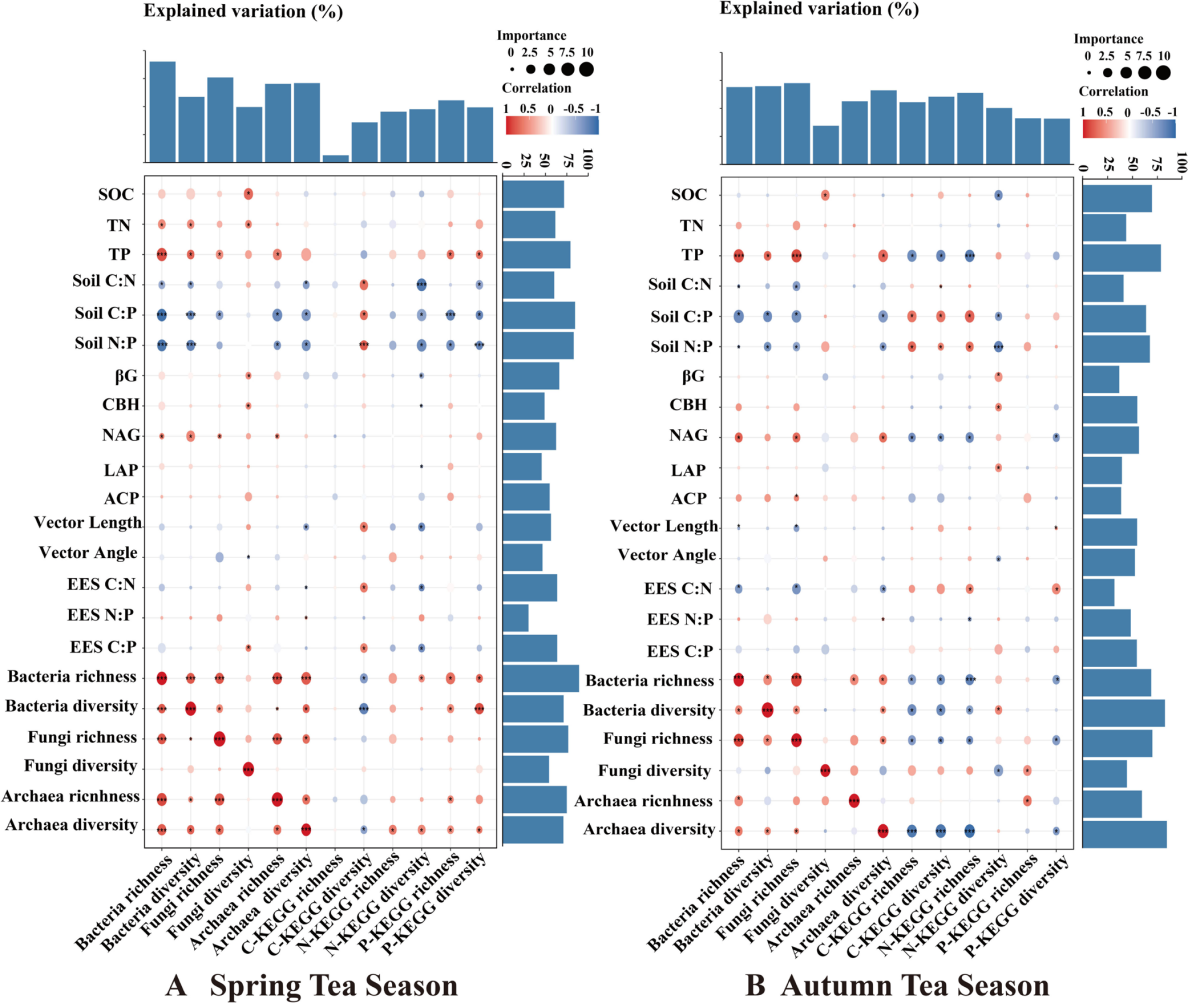
Heat map of the most important predictors of soil microbial and functional gene abundance and diversity in tea plantations during early (April) and late (August) tea picking of categories based on the random forest analysis. Circle size corresponds to the variable importance; blue and red colors reflect negative and positive Spearman correlations, respectively.

**FIGURE 6:**
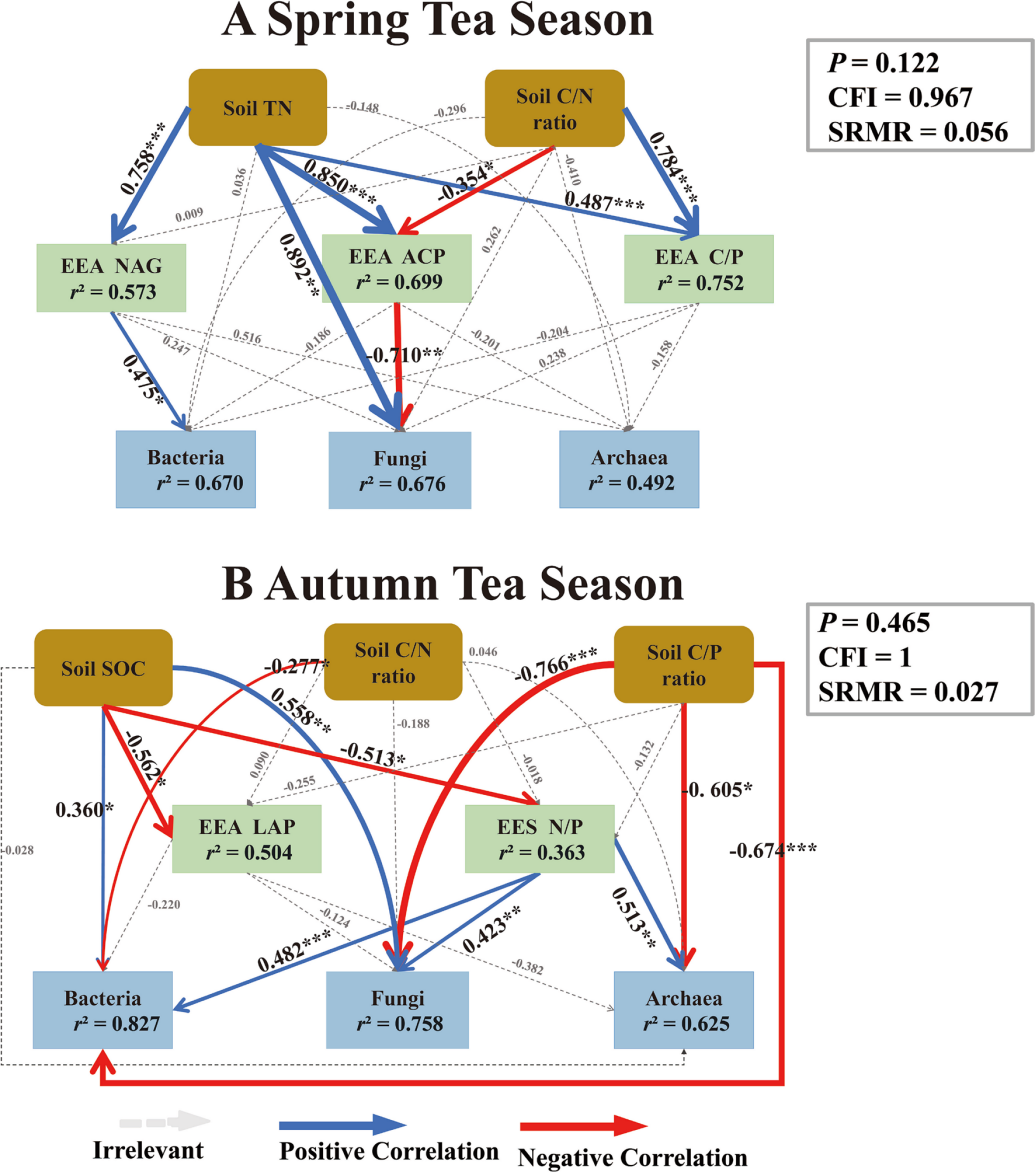
SEM of the relationships between soil microbial communities with soil physicochemical properties and extracellular enzyme activities in tea plantations (*p* = 0.236, CFI > 0.90, SRMR < 0.08), with the blue line to be changed to a positive correlation, the red line to represent a negative correlation, and the gray dotted line to represent no significant correlation.

The correlation analysis revealed that soil microorganisms (bacteria, fungi, and archaea) and soil functional genes (C‐KEGG, N‐KEGG, and P‐KEGG) were strongly correlated with the selected soil biogeochemical indicators (soil physicochemical factors, soil C:N:P, EEA, EES C: N:P, and VIF < 10) (Figures 3, 4, 5, 6).

#### 3.5.1 Mantel Test Analyses

Mantel tests revealed that in spring, soil bacteria and N‐KEGG presented strong correlations with the soil C:N, N:P, LAP, and EES C:P ratios (*p* < 0.05). Fungal communities were significantly correlated with TN, C:N, N:P, CBH, and EES C:P (*p* < 0.05). Archaea were significantly linked to the soil C:N, N:P, CBH, LAP, and EES C:P ratios. C‐KEGG and P‐KEGG were significantly associated with the soil C:N, N:P, and EES C:P ratios. In autumn, the TP ratio was significantly correlated with all the soil microorganisms and functional genes (*p* < 0.05). The EES C:N ratio was significantly associated with bacteria, fungi, and functional genes (*p* < 0.05) but not with archaea (Figure 3, Table S6).

#### 3.5.2 Redundancy Analysis (RDA)

To elucidate the relationships among the soil microbial communities, functional genes, and soil biogeochemical indicators, redundancy analysis (RDA) was conducted (Figure 4, Table S7). In spring, 64% and 82.9% of the variance in the soil microbial communities and functional genes, respectively, was explained by the environmental variables. Most soil factors (TN, N:P, CBH, LAP, and EES C:P) were significantly correlated with both microbial communities and functional genes (*p* < 0.05), except for the soil C:N and EES N:P ratios (Table S7). In autumn, the soil biogeochemical indicators explained 67.3% and 78.3% of the variance in the microbial communities and functional genes, respectively. The TP content was strongly correlated with microbial communities and functional genes, whereas the EES C:N ratio was significantly associated with functional genes but not with archaeal communities (*p* < 0.05).

#### 3.5.3 Random Forest (RF) Analysis

**FIGURE 7:**
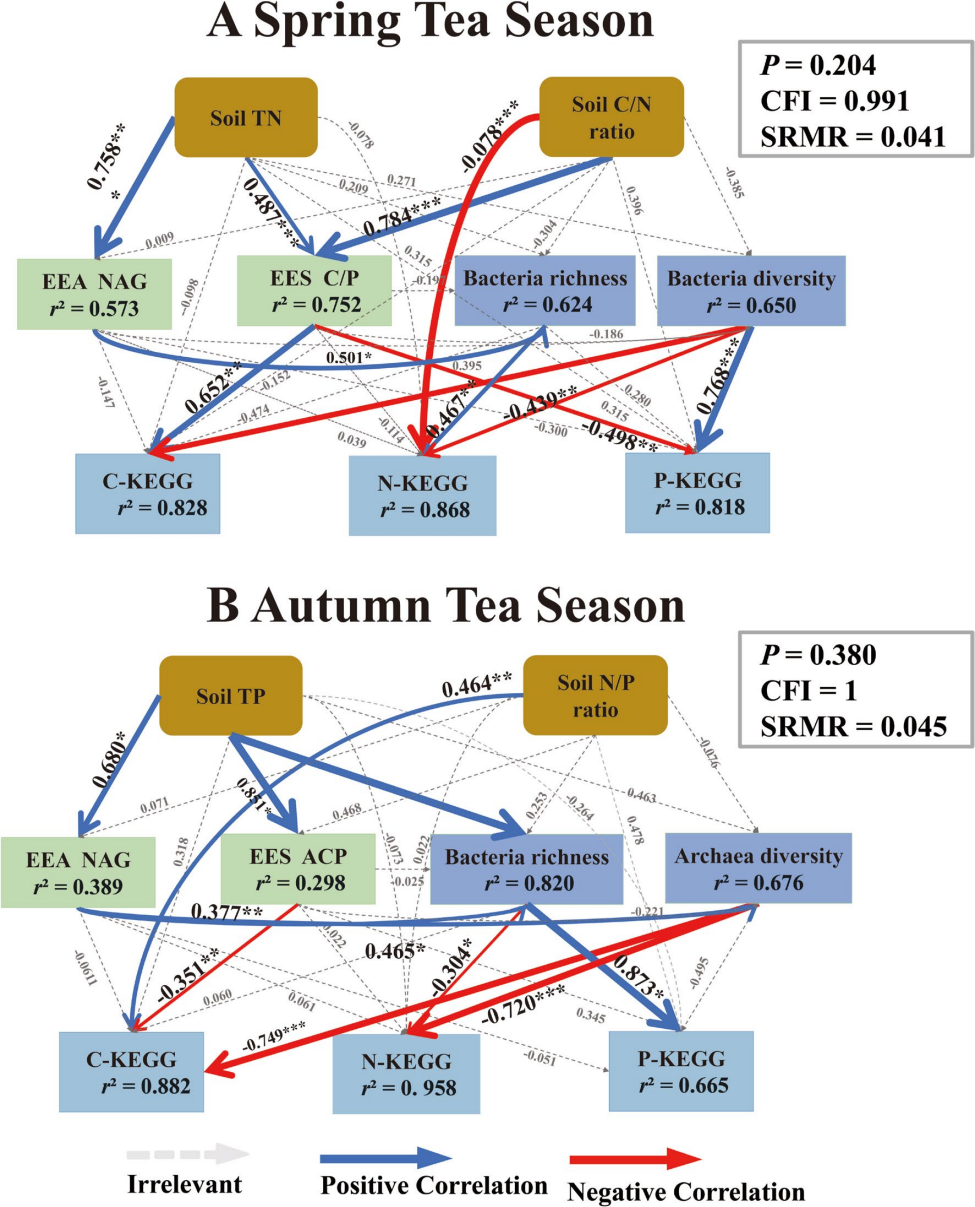
SEM of the relationships between soil functional genes with soil physicochemical properties and extracellular enzyme activities in tea plantations (*p* = 0.236, CFI > 0.90, SRMR < 0.08), with the blue line to be changed to a positive correlation, the red line to represent a negative correlation, and the gray dotted line to represent no significant correlation.

Random forest (RF) analysis (Figure 5, Table S8) revealed that TP, the soil N:P ratio, and the C:P ratio are the most critical predictors of soil microbial communities and functional gene dynamics across seasons (*R* ^2^ > 0.5). Bacterial richness and diversity, alongside archaeal diversity, were critical predictors of functional gene profiles (*R* ^2^ > 0.7). Notably, the SOC and EES C:N ratios were specifically linked to fungal community variation, although the EES C:N ratio exhibited weaker explanatory power (Figure 7, *p* < 0.05). In spring, βG, soil C, and ACP further contributed to microbial and functional gene variability (*R* ^2^ > 0.5). The phosphorus‐related metrics (TP, N:P, and C:P) consistently outperformed the nitrogen‐associated variables (TN and EES N:P) in terms of explanatory power (*R* ^2^ > 0.5 vs. < 0.48). In autumn, additional significant predictors included NAG, CBH, EEA vector length, EES C:P, and EEA vector angle (*R* ^2^ > 0.5).

#### 3.5.4 Structural Equation Modeling (SEM)

This study utilized a structural equation modeling (SEM) (Figures 6 and 7) with the best fit (*p* > 0.05, CFI > 0.90, RMSEA < 0.08) to further explain the direct and indirect effects of soil factors on soil microorganisms and soil functional genes, thereby providing a systematic understanding of the driving factors of changes in soil microorganisms and soil functional genes. In spring, the soil TN, C:P, and EES C:P ratios had strong direct effects on the microbial communities (Figure 6A, Figure S6A). βG and ACP modulated carbon and phosphorus acquisition pathways, whereas bacterial richness and diversity directly enhanced functional gene diversity (C‐KEGG, N‐KEGG, P‐KEGG; Figure 7A, Figure S7A). During autumn, TP, SOC, and phosphorus‐related stoichiometric ratios (C:P, N:P) had both direct and indirect effects on microbial communities (Figure 6B, Figure S6B). Extracellular enzyme activities (NAG, LAP, and ACP) and EES N:P further mediated nutrient cycling processes, with bacterial richness and archaeal diversity directly driving functional genes (Figure 7B, Figure S7B).

## 4 Discussion

### 4.1 Interactions Between Soil Physicochemical Properties and the Dynamics of Extracellular Enzyme Activities: Effects of Agricultural Practices and Seasonal Variations

**FIGURE 8:**
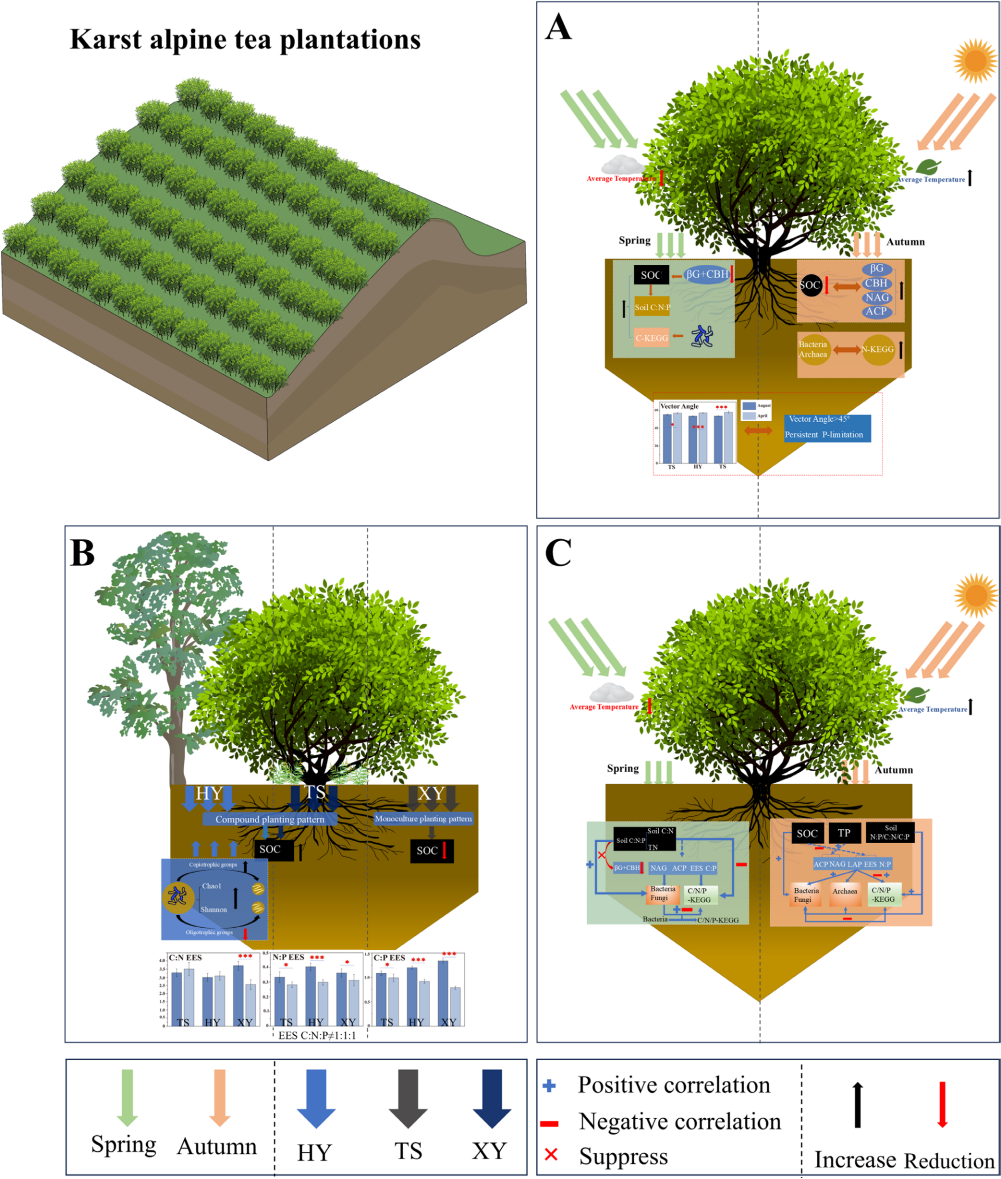
A comprehensive framework for seasonal drivers and management strategies for Karst alpine tea plantations. (A) is the seasonal driver (spring and autumn); (B) is for management practices (organic (HY), pollution‐free (TS), and conventional (XY) practices); and (C) is the ecological relationship.

This study investigated the impacts of seasonal variations and agricultural management on soil ecosystem functions in karst tea plantations, focusing on nutrient dynamics (SOC, TN, and TP) and extracellular enzyme activities (EEA) (Figure S1, Table S2). Key findings reveal that SOC exhibits significant seasonal declines in autumn (*p* < 0.05) due to thermally accelerated microbial decomposition (Zheng et al. 2020) (Figure 8A). In contrast to conventional monocultures, organic and pollution‐free management practices—characterized by diversified cropping—maintain higher SOC levels. This aligns with agroecological strategies for preserving organic matter (Wang and Cao 2011). Enzymatic activity is suppressed under cold or low‐humidity conditions as well as excessive moisture or elevated temperatures (Zheng et al. 2020). These observations are consistent with the elevated EEA recorded in autumn during our study. The seasonal contrast is further explained by two synergistic mechanisms: increased litterfall inputs during peak tea harvesting and management interventions such as organic mulching and selective pruning. Collectively, these results highlight the dual regulatory roles of climatic variability and anthropogenic management in soil biogeochemical cycles. This underscores the need for seasonally adaptive strategies to ensure sustainable management of fragile karst agroecosystems.

The soil C:N:P ratio exhibits a strong dependence on both season and management. Elevated ratios in spring correlate with regional temperature, precipitation, and pedogenic processes (Tan et al. 2021). Organic management (HY) effectively lowers C:N ratios through controlled residue inputs and enhanced nitrogen retention via organic amendments (Bai et al. 2018). Contrary to the theoretical 1:1:1 extracellular enzyme stoichiometry (EES C:N:P) expected under microbial homeostasis (Sinsabaugh and Follstad Shah 2012), karst soils display resource‐driven stoichiometric patterns (Figure 8B, Table S2). This supports the substrate availability hypothesis in ecosystems with variable resources (Peng and Wang 2016). This deviation stems from geochemical constraints: calcium–magnesium interactions promote phosphorus fixation through mineral adsorption (Yan et al. 2018), thereby limiting microbial phosphorus availability. In response, microbial communities employ adaptive strategies including increased acid phosphatase activity (ACP; Figure S1) and reduced EES C:P ratios. These mechanisms mirror those observed in phosphorus‐limited agricultural systems (Cui et al. 2020; Zhang, Ai, et al. 2019; Zhang, Xu, et al. 2019). Seasonal variation significantly influences EES patterns (*p* < 0.05), with autumn increases associated with labile litter inputs (Wittmann et al. 2004). Critically, vector angles > 45° and EES N:P < 1 across all sites confirmed pervasive phosphorus limitation (Cui et al. 2020), contradicting our initial hypothesis (H1) that seasonal variations insignificantly affect metabolic constraints. This corroborates reports of seasonally modulated phosphorus limitation in subtropical agroecosystems (Guan et al. 2020). Future studies should combine long‐term monitoring with metagenomic approaches to quantify microbial metabolic remodeling under resource fluctuations, enabling precise predictions of karst ecosystem resilience.

### 4.2 Structural and Functional Differences in Soil Microbial Communities and Functional Genes: Revealing Responses to Management Practices and Seasonal Variations

Soil microbial communities critically regulate terrestrial nutrient cycling in karst alpine tea ecosystems, although their regulatory mechanisms remain poorly characterized (Rui et al. 2015). Our findings demonstrate that organic management enhances microbial diversity through niche partitioning, promoting copiotrophic groups (Proteobacteria/Actinobacteria) over oligotrophic groups (Acidobacteria/Verrucomicrobia) under resource‐enriched conditions (Liao et al. 2023). Seasonal analyses revealed that prokaryotic richness significantly increased from spring to autumn, which aligns with microbial sensitivity to temperature and moisture gradients (Fierer 2017; Han et al. 2021) (Figure 8B, Table S3). Functional gene profiling revealed three key patterns: (1) spring carbon fixation genes (C‐KEGG) peak in richness (Chao1) and diversity (Shannon), which correlate with microbial adaptation to low temperatures via preferential assimilation of labile carbon from SOC deposition (Domeignoz‐Horta et al. 2020) (Tables S2 and S5); (2) autumn nitrogen cycling gene (N‐KEGG) abundance increases with temperature‐enhanced ammonia oxidation, in contrast with spring precipitation‐induced denitrification suppression (Tang et al. 2019); and (3) management practices amplify the seasonality of phosphorus cycling genes (P‐KEGG), suggesting that the timing of fertilization regulates functional expression (Ma et al. 2021) (Table S5). In summary, seasonal climatic fluctuations—rather than management practices—emerged as the primary driver of microbial functional gene dynamics. This finding contrasts with Hypothesis H2, which posited that management practices would dominate regulatory effects. Although organic management enhanced microbial diversity, its dependence on diversified resource inputs (e.g., intercropping, organic amendments) and interactions with seasonal climatic variations may have reinforced the dominance of seasonal fluctuations in functional gene dynamics. This aligns with the finding that climatic factors, rather than management practices, primarily drove microbial functional patterns (Tables S4 and S5). Therefore, we suggest optimizing spring nitrogen and phosphorus fertilizer management, reducing autumn disturbance to maintain microbial functional stability, and focusing on the sensitive response of nitrogen and phosphorus cycling genes to provide a scientific basis for the ecological management of tea plantations.

### 4.3 Interwoven Networks: Linking Soil Biogeochemical Drivers, Extracellular Enzymes, and Microbial Functional Potentials

Elevated soil C:N:P ratios in spring intensified microbial competition for nutrients. This led to stronger correlations between environmental variables (e.g., TN; soil C:N ratio) and microbial communities or functional genes. This intensified competition may further suppress enzymatic activities (e.g., βG and LAP; Table S2), likely driven by seasonal variations in soil nutrient availability and litter inputs, which alter microbial resource allocation (Bardgett et al. 2005; Liu et al. 2016). In autumn, however, increased litterfall inputs provide labile organic carbon (Bai et al. 2024). This rapidly stimulates bacterial and fungal growth, alongside enzymatic decomposition (e.g., CBH; Table S2). However, temperature‐enhanced microbial activity combined with preferential labile carbon utilization accelerated SOC mineralization. Consequently, net SOC decreased (Table S2). Archaea exhibit weaker responses to labile carbon inputs due to their specialization in recalcitrant carbon metabolism (Baker et al. 2020). This may exacerbate carbon loss via methane emissions. The negative soil C:P–microbial community correlation reflects adaptive mechanisms. These include increased phosphatase activity (Ragot et al. 2017) and enrichment of phosphate‐solubilizing microbes (Cui et al. 2020). Agricultural management practices induce functional trade‐offs. Compared with conventional monocultures (XY), organic management (HY) maintains functional redundancy through diverse substrate inputs (Lori et al. 2023), increasing phosphorus metabolic efficiency and buffering stoichiometric imbalances. Bacterial diversity is a key predictor of functional gene variation. This likely stems from its dominance in carbon/nitrogen cycling (Luo et al. 2020) and adaptability to resource fluctuations (Sinsabaugh et al. 2008). In contrast, conventional practices result in greater stability under reduced disturbance (Dai et al. 2020) but lower functional plasticity, rendering them vulnerable to seasonal extremes (Bahram et al. 2022) (Figure 8C). Based on these findings, we propose three targeted strategies: (1) optimize C:N during spring by supplementing carbon sources (e.g., straw) to regulate microbial metabolism; (2) autumn organic phosphorus fertilization (e.g., composted rock phosphate) to alleviate P limitation, mimicking strategies validated in citrus orchards (Ye et al. 2022; Han et al. 2024); and (3) introduction of crops for intercropping and enrichment of phosphorus‐solubilizing microorganisms through root secretions (Zhou and Chen 2019) (Table S9). These strategies may increase microbial resilience to climatic fluctuations and improve ecosystem sustainability.

## 5 Conclusions

In conclusion, seasonal variations and agricultural practices jointly regulate soil properties and enzyme activities in karst alpine tea plantations, driving microbial adaptation to phosphorus limitation. Elevated C:N:P ratios in spring suppressed enzyme activity (e.g., βG and LAP), whereas autumn litter inputs increased bacterial dominance. The soil EES C:N:P ratio stoichiometry in alpine tea plantations in karst areas depends on soil resource effectiveness rather than dynamic equilibrium. Bacterial diversity governs functional genes, with organic management enhancing resilience via functional redundancy. We propose the following: (1) optimize C:N during spring by supplementing carbon sources (e.g., straw) to regulate microbial metabolism; (2) autumn organic phosphorus fertilization (e.g., composted rock phosphate) to alleviate P limitation, mimicking strategies validated in citrus orchards; and (3) introduction of crops for intercropping and enrichment of phosphorus‐solubilizing microorganisms through root secretions (Table S9). Future studies should integrate multiomics approaches and conduct long‐term experimental monitoring to validate gene and observed stoichiometric pattern linkages. These efforts will advance precision management in karst ecosystems, offering a sustainable agricultural blueprint in fragile environments.

## Author Contributions

**Tianyi Pu:** conceptualization (equal), data curation (equal), formal analysis (equal), investigation (equal), methodology (equal), resources (equal), software (equal), supervision (equal), validation (equal), visualization (equal), writing – original draft (equal), writing – review and editing (equal). **Yusha Tan:** conceptualization (equal), formal analysis (equal), investigation (equal), methodology (equal), resources (equal), software (equal), validation (equal), visualization (equal). **Yuanqi Zhao:** data curation (equal), investigation (equal), resources (equal), software (equal), validation (equal), visualization (equal). **Zhibin Zhao:** conceptualization (equal), data curation (equal), investigation (equal), resources (equal), supervision (equal), validation (equal), visualization (equal). **Ni Zhang:** conceptualization (equal), data curation (equal), formal analysis (equal), investigation (equal), resources (equal), supervision (equal), visualization (equal). **Can Li:** conceptualization (equal), data curation (equal), investigation (equal), project administration (equal), resources (equal), validation (equal), visualization (equal). **Yuehua Song:** formal analysis (equal), funding acquisition (equal), methodology (equal), project administration (equal), supervision (equal), validation (equal), writing – review and editing (equal).

## Disclosure

*Statement on inclusion:* Our study fails to include scientists based in the country where the study was carried out. We recognize that more could have been done to engage local scientists with our research as our project developed and to embed our research within the national context and research priorities. We are planning to address these caveats in future research.

## Ethics Statement

The authors have nothing to report.

## Conflicts of Interest

The authors declare no conflicts of interest.

## Supporting information


Figure S1

Figure S2

Figure S3

Figure S4

Figure S5

Figure S6

Figure S7

Table S1

Table S2

Table S3

Table S4

Table S5

Table S6

Table S7

Table S8

Table S9

## Data Availability

All raw data generated in this study have been uploaded to the NCBI BioProject database under accession number PRJNA1152139. All the data analysis results obtained during this study are included in the manuscript and its Supporting Information.
