# Supplementary material for: Effect of Seasonal Variations on Soil Microbial, Extracellular Enzymes, and Ecological Stoichiometry in Tea Plantations
Source: Ecol Evol. 2025 May 12;15(5):e71362. doi: 10.1002/ece3.71362 (PMC12069803; doi:10.1002/ece3.71362)
Supplement: Supplementary file 2 — Figure S2 [file ECE3-15-e71362-s015.docx]

Figure S2. Cricos plots display species with abundances greater than or equal to 0.01 (species level), while bar graphs represent the top 10 abundances at the gate level. Figures A, B, and C depict bacteria, fungi, and archaea for August, respectively, and Figures D, E, and F depict bacteria, fungi, and archaea for April, respectively.
