# Supplementary material for: Effect of Seasonal Variations on Soil Microbial, Extracellular Enzymes, and Ecological Stoichiometry in Tea Plantations
Source: Ecol Evol. 2025 May 12;15(5):e71362. doi: 10.1002/ece3.71362 (PMC12069803; doi:10.1002/ece3.71362)
Supplement: Supplementary file 3 — Figure S3 [file ECE3-15-e71362-s007.docx]

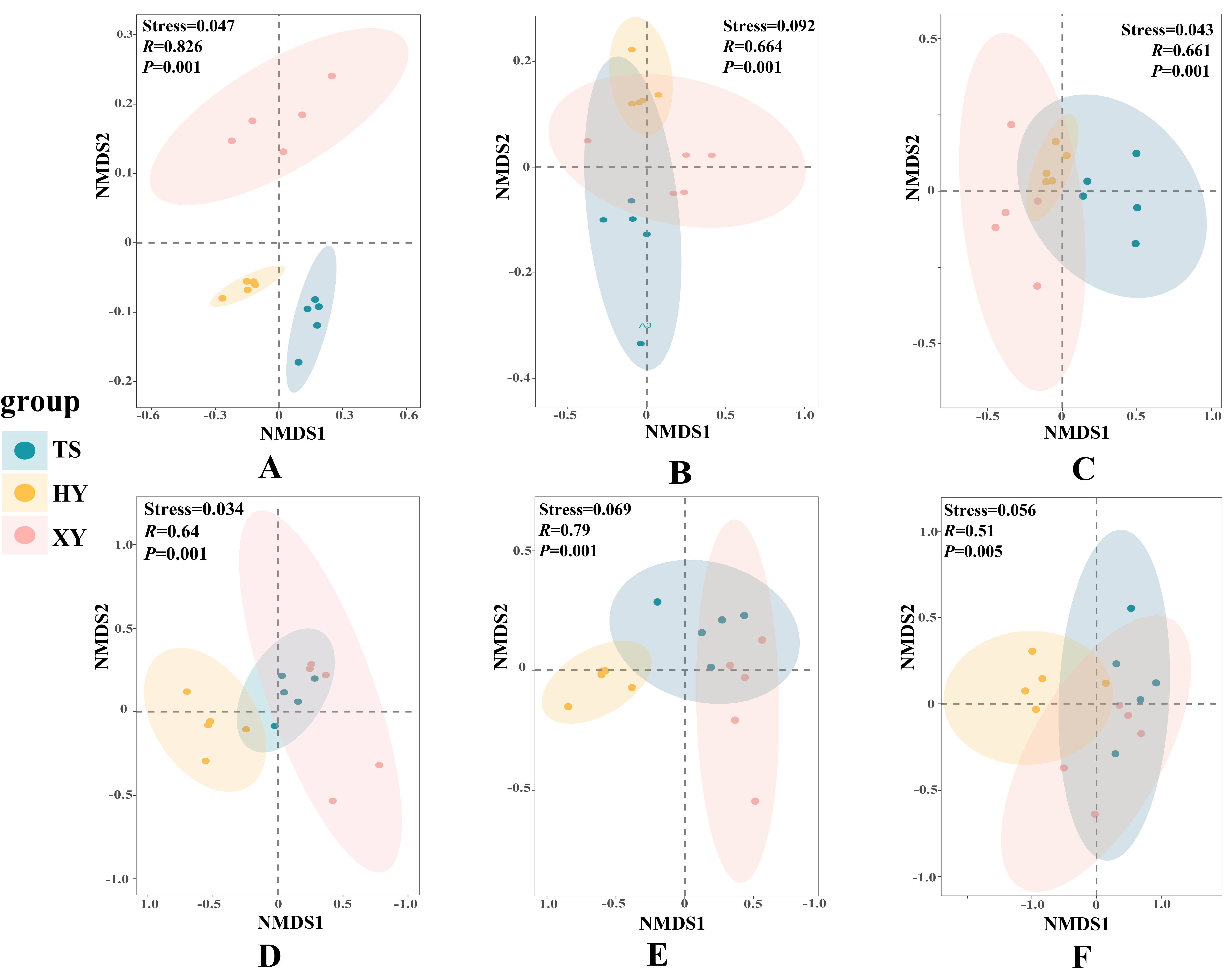


**Figure S3.** NMDS analysis based on Bray-Curtis distance matrix, Figures A, B, and C are the bacterial fungal and archaeal communities of three tea plantations collected in spring, and Figures D, E, and F are the bacterial, fungal, and archaeal communities of three tea plantations collected in autumn, respectively.
