# Supplementary material for: Effect of Seasonal Variations on Soil Microbial, Extracellular Enzymes, and Ecological Stoichiometry in Tea Plantations
Source: Ecol Evol. 2025 May 12;15(5):e71362. doi: 10.1002/ece3.71362 (PMC12069803; doi:10.1002/ece3.71362)
Supplement: Supplementary file 4 — Figure S4 [file ECE3-15-e71362-s010.docx]

**Figure S4.** Cricos plots display KEGGs with a combined abundance greater than or equal to 0.01, while bar graphs illustrate the top 10 abundances at pathway level 3 (pathway ID). Figures A, B, and C represent C-KEGG, N-KEGG, and P-KEGG for August, respectively, and Figures D, E, and F represent C-KEGG, N-KEGG, and P-KEGG for April, respectively.
