# Supplementary material for: Effect of Seasonal Variations on Soil Microbial, Extracellular Enzymes, and Ecological Stoichiometry in Tea Plantations
Source: Ecol Evol. 2025 May 12;15(5):e71362. doi: 10.1002/ece3.71362 (PMC12069803; doi:10.1002/ece3.71362)
Supplement: Supplementary file 5 — Figure S5 [file ECE3-15-e71362-s005.docx]

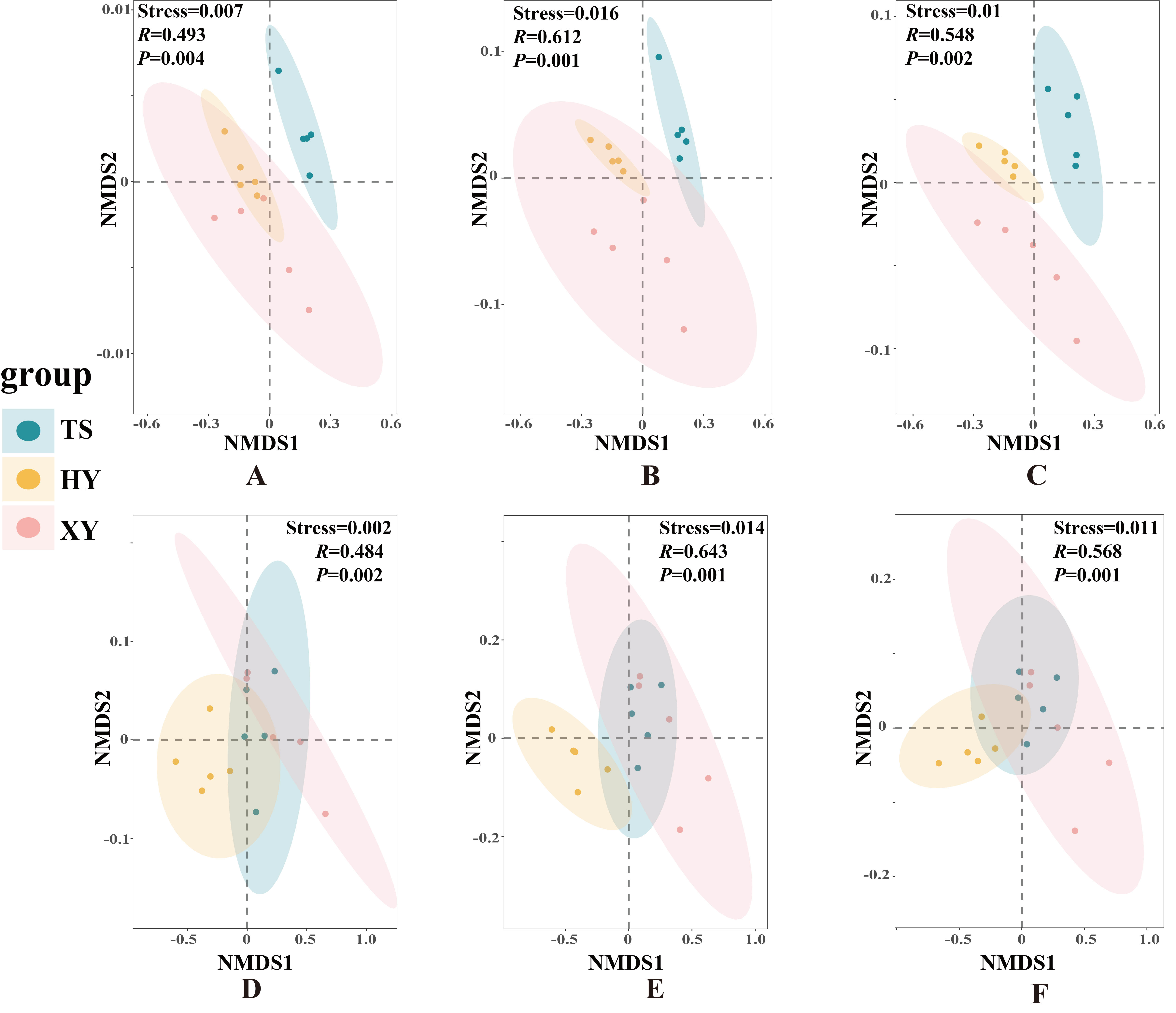


**Figure S5.** NMDS analysis based on Bray-Curtis distance matrix and ANOSIM analysis, Figures A, B, and C are C-KEGG, N-KEGG, and P-KEGG in spring, respectively; and Figures D, E, and F are C-KEGG, N-KEGG, and P-KEGG in autumn, respectively.
