# Supplementary material for: Effect of Seasonal Variations on Soil Microbial, Extracellular Enzymes, and Ecological Stoichiometry in Tea Plantations
Source: Ecol Evol. 2025 May 12;15(5):e71362. doi: 10.1002/ece3.71362 (PMC12069803; doi:10.1002/ece3.71362)
Supplement: Supplementary file 6 — Figure S6 [file ECE3-15-e71362-s002.docx]

**Figure S6.** Effect values for structural equations, including direct effects, indirect effects and total effects (microorganisms)
